# Modeling pulsativity in the hypothalamic–pituitary–adrenal hormonal axis

**DOI:** 10.1038/s41598-022-12513-w

**Published:** 2022-05-19

**Authors:** Alexander N. Churilov, John G. Milton

**Affiliations:** 1 Faculty of Mathematics and Mechanics, Saint Petersburg State University, Saint Petersburg, Russia; 2 W. M. Keck Science Center, The Claremont Colleges, Claremont, CA USA

**Keywords:** Neuroscience, Endocrinology, Engineering

## Abstract

A new mathematical model for biological rhythms in the hypothalamic–pituitary–adrenal (HPA) axis is proposed. This model takes the form of a system of impulsive time-delay differential equations which include pulsatile release of adrenocorticotropin (ACTH) by the pituitary gland and a time delay for the release of glucocorticoid hormones by the adrenal gland. Numerical simulations demonstrate that the model’s response to periodic and circadian inputs from the hypothalamus are consistent with those generated by recent models which do not include a pulsatile pituitary. In contrast the oscillatory phenomena generated by the impulsive delay equation mode occur even if the time delay is zero. The observation that the time delay merely introduces a small phase shift suggesting that the effects of the adrenal gland are “downstream” to the origin of pulsativity. In addition, the model accounts for the occurrence of ultradian oscillations in an isolated pituitary gland. These observations suggest that principles of pulse modulated control, familiar to control engineers, may have an increasing role to play in understanding the HPA axis.

## Introduction

The hypothalamic–pituitary–adrenal (HPA) axis plays a central role in coordinating the neuroendocrine adaptation of the stress response^1^ and in the synchronization of peripheral circadian clocks located downstream to the suprachiasmic nucleus (SCN)^2,3^. Consequently this axis forms the substrate upon which the central nervous system interacts with the hormonal endocrine system. Mathematical modeling has played an important role in uncovering the nature of this interaction^3–6^. However, new experimental observations typically necessitate the formulation of new models. Historically mathematical models of the HPA axis took the form of ordinary differential equations formulated using the laws of mass action^7,8^. More recently, the presence of significant time delays ($\sim 15$ min) has been recognized. This time delay is thought to arise because of the time required for the synthesis and release CORT into the blood stream. This observation, in turn, has led to models formulated in terms of delay differential equations^8–11^. Here we take into account the observation that inputs arising from the nervous system^12^ and the pituitary gland^13^ are discrete, or pulsatile, in nature. Pulsativity represents a strong non-linearity and is expected to have a significant impact on the dynamics. Given the impulsive nature of signalling within the HPA axis^14^ it is natural to apply the principles of pulse modulated control^15^.

**Figure 1:**
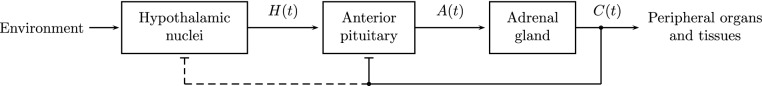
Schematic representation of the HPA axis studied in this paper. Arrows and bar-headed lines indicate excitatory and inhibitory connections, respectively. Following^16^, the feedback shown by the dashed line is neglected.

The HPA system (Fig. 1) is often considered to be a prototypical example of a physiological feedback control mechanism^17–19^. There are three, spatially separated components: the hypothalamus, the anterior pituitary gland, and the adrenal cortex. The three main hormones involved in the HPA control loop are corticotropin-releasing hormone (CRH) produced by the neurons of the paraventricular nucleus (PVN) of the hypothalamus, adrenocorticotropin (ACTH) secreted by the corticotroph cells of the anterior pituitary, and glucocorticoid hormones (CORT), predominantly cortisol in humans and corticosterone in rodents, produced by the adrenal gland^20^. The CRH concentration in blood induces secretion of ACTH, which in its turn activates synthesis and secretion of CORT. CORT has negative feedback effect on the secretions to the bloodstream of ACTH and CRH^16,18,21,22^.

HPA is characterized by complex, oscillatory dynamics. The main hormonal rhythms observed are ultradian (with a period $\sim 1$ h) and circadian (with a period $\sim 24$ h)^23,24^. The circadian hormonal release is modulated by the external pacemaker lying in the suprachiasmatic nucleus (SCN) of hypothalamus. The SCN receives a light/dark information from the environment and is not involved in the regulation loop. In contrast, the ultradian rhythm is an important and intrinsic property of the HPA control system^16,25^. The pulsatile cellular activity in the pituitary gland is a key regulator of hormone secretion^26–28^. However, the existence of a hypothalamic CRH pulse generator has also been confirmed in vitro^29^. Thus it is possible that the pituitary and hypothalamic pulse generators coexist and somehow interact.

Both of the hypothalamo-pituitary hormones CRH and ACTH are released as pulses^30,31^. Corticoid hormones are released more smoothly, but inherit a pulsatile pattern from CRH and ACTH^31^. For a number of years it was assumed that the center of ultradian pulsativity of HPA was the hypothalamic nucleus (see e.g.^31,32^). Although this hypothesis was confirmed for the reproductive and growth hormones, it was disproved for the HPA axis (see a discussion in^33^). It was shown in vivo that an ultradian CORT rhythm exists, even when the hypothalamus is surgically disconnected from the pituitary^34^. Another reason to doubt the leading role of the hypothalamic frequency is that the frequency of the CRH release was found to be three times higher than that of ACTH^30,35^. Finally it has been shown that ultradian oscillations in CORT persist in the presence of a constant level of CRH^36^. Taken together these observations strongly indicate that the pulse generator in the HPA axis is sub-hypothalamic.

Our discussion is organized as follows. First, we build our model upon realistic mathematical models developed previously for the HPA axis which emphasize that pulsatile glucocorticoid production arises due to a sub-hypothalamic pulse generator as a result of the interplay between the pituitary and the adrenal gland^8,11,16,20,36–39^. An integrate-and-fire mechanism is used to illustrate the effects of pulsativity of the anterior pituitary gland. Then, using numerical simulations, we demonstrate that this model reproduces the experimentally observed patterns of ultradian and circadian oscillations as the hypothalamic input to the pituitary gland is varied. Next we show that, in contrast to previous models^8,16^, the time delay is not critical for oscillatory behavior, but merely introduces a phase shift. Finally, we show how ultradian oscillations can arise in an isolated pituitary gland, i.e. when the pituitary is disconnected from both of the hypothalamus and the adrenal gland^34^. Notice that if the level of CORT is zero, the pituitary-adrenal pulse generator proposed in^16^ does not induce periodic oscillations of ACTH, which were observed in vitro in an isolated human pituitary^34^. Despite the simplicity of our model, it not only captures the salient features of the ultradian and circadian rhythms generated by the HPA axis, but also explains previously unexplained observations.

## Model

### Background

Our model builds on a realistic mathematical model for the HPA ultradian pulsativity proposed in^8,11,16,20,36–39^. Following Walker et al.^16^ we assume: (1) the CRH level is constant during the system’s transient time; (2) the impact of glucocorticoids on the CRH production is not important for the system’s dynamics and can be neglected; (3) a discrete time delay was introduced in the term describing the CORT production. It was shown that for some physiologically reasonable values of the CRH constant level and of the delay this modified 3D system exhibited periodic oscillations that could be interpreted as ultradian; (4) When the hypothalamic input is periodic, a realistic circadian rhythm is obtained.

The mathematical model that we propose has the following significant distinctions from the previously known models: (1) describing the corticotroph pulse generator with the help of an electrical action potentials was previously proposed in^40,41^. However these works used a Hodgkin–Huxley-like formalism, which is much more complicated and employs more equations than the integrate-and-fire formalism that we use; (2) the models based on^16,22^ contain a nonlinear equation that describes dynamics of glucocorticoid receptors (GR) in the pituitary. Our model is more parsimonious and does not consider GR; (3) in case the inputs from the adrenal gland and from the hypothalamus to the pituitary are lacking, the concentration of ACTH vanishes in the GR-oriented models. In our model oscillations of ACTH are supported even for zero inputs; (4) in GR-oriented models oscillativity of the hormone concentrations is attained by the introduction of a time delay into the equation describing the CORT release. Our model remains oscillative without such delay.

### Additional assumptions

We make the following additional assumptions: (1) The center of HPA ultradian pulsativity is located in pituitary^8,11,16,36,42^; (2) ACTH and CORT are released in pulses with an ultradian frequency of approximately one pulse per hour^8^; (3) The pituitary hormonal cells generate some membrane electrical potential, just like neurons, and this potential controls the amount of released ACTH^43,44^; (4) The pre-synthesized ACTH is accumulated in secretory vesicles near the cell membrane. It is released very rapidly in response to hypothalamic stimulation^8^; (5) When ACTH reaches the adrenal gland, it launches the process of CORT synthesis. Thus, unlike ACTH, CORT cannot be released immediately, but requires some time for synthesis^9^. Following^8^, we took this circumstance into account by choosing the time delay of 15 min in the feedforward connection between the pituitary and the adrenal gland; (6) The level of CORT follows the circadian profile. It is high during the active time and low in the sleep period. A disruption of the circadian rhythm nevertheless preserves the ultradian rhythm^34,45^; (7) The release of CRH is also pulsatile with an approximate frequency of three pulses per hour. While the CRH pulse frequency is rather steady during the diurnal period, the amplitudes of CRH pulses vary significantly, following the circadian rhythm^30,42^; (8) The increase of the constant CRH level implies an increase of the ultradian frequency^8^. The blockade of endogenous CRH results in a significant reduction of the amplitudes of ultradian pulses^42^. (9) CORT negative feedback at the hypothalamus is not an important factor in regulating the dynamic activity of the HPA system, so this feedback can be neglected^16^; (10) We neglect the existence of extra-pituitary mechanisms for ACTH secretion^46^.

### Mathematical formulation

Following the assumptions of the previous section, we suggest the following mathematical model. Consider a system of differential equations with delay. The two main variables involved are *A*(*t*) and *C*(*t*) that represent concentration levels of ACTH and CORT in blood, respectively.

1. Equation (1) relates to the adrenal gland and follows^16^. The concentration *C*(*t*) satisfies a differential equation with delay
  1 $$\begin{aligned} {\dot{C}} = -k_c C + k_{ca} A(t-\tau ), \end{aligned}$$
  where $k_c$ is a degradation rate of CORT and the coefficient $k_{ca}$ relates to the driving (feedforward) signal from the pituitary to the adrenal gland. The delay $\tau$ reflects the time required for the CORT synthesis.
2. Equations (2)–(5) describe the dynamics of the ACTH release by the corticotropic cells in the anterior pituitary. The spiking dynamics of these cells are quite complex and include spiking, bursting and irregular spiking patterns. These complexities arise because of the effects of BK (big potassium) channels on the dynamics of conductance-based models (see Discussion). Here we make the simplifying assumption that the pituitary release of ACTH can be described by an integrate and fire mechanism. The pulse generator located in the anterior pituitary is modeled with two equations, the first of them is ordinary differential and the second one is a differential equation with jumps (impulses):
  2 $$\begin{aligned} {\dot{A}}= -k_a A + ( A_0 + k_{ah} H)\, F_a(C) F_v(V), \end{aligned}$$
  3 $$\begin{aligned} {\dot{V}}= -k_v V + (V_0 + k_{vh} H)\,F_a(C). \end{aligned}$$
  The variable *V*(*t*) is a charging-discharging membrane potential related to pituitary cells (cf.^44^). The pulsation times are defined from the recursion
  4 $$\begin{aligned} t_0=0, \quad t_{n+1} = \min \{t \;:\; t>t_n, \quad V(t)=\Delta \}, \end{aligned}$$
  where $\Delta$ is a given firing threshold. After the firing the potential *V*(*t*) resets to zero:
  5 $$\begin{aligned} V(t_n^+) = 0. \end{aligned}$$
  Here $V(t_n^+)$ is the right-sided limit of *V*(*t*) at the point $t_n$. Together (3)–(5) implement an integrate-and-fire scheme with a leakage coefficient $k_v$. a threshold $\Delta$ and an excitatory input
  $$\begin{aligned} I(t) = (V_0 + k_{vh} H(t))\,F_a(C(t)) \end{aligned}$$
  (cf.^44^). The greater is *I*(*t*), the faster *V*(*t*) reaches the threshold, so the firing frequency increases. If the threshold is never reached, there will be no impulses (the system approaches an equilibrium). All the jumps of *V*(*t*) are of the same value (equal to $\Delta$).

The function $F_a(C)$ describes a feedback from CORT to ACTH and *V*. It is decreasing in *C* to ensure an inhibition of the ultradian amplitude and frequency when the level of CORT increases. With the increase of *C*(*t*) the right-hand side of (2) and the excitatory input *I*(*t*) decrease, so the level of *A*(*t*) also decreases (is inhibited) and the impulses of *V*(*t*) become sparser. In our simulations the function $F_a(C)$ is taken to represent repression Michaelis—Menten kinetics

6 $$\begin{aligned} F_a(C) = F_0+ \frac{1}{1+ C/h_c}, \end{aligned}$$

where $F_0$, $h_c$ are some positive parameters.

Notice that the presence of feedback $F_a(C)$ is not critical for oscillativity of ACTH. Namely, if we neglect the inhibitory effect of CORT by setting $F_a(C)\equiv F_0={\text{const}}$, the function *A*(*t*) still may oscillate. The main role of this feedback is an additional (to the hypothalamic input) modulation of amplitudes and frequencies.

The non-linearity $F_v(V)$ is empirical, it relates to the shape of a single ACTH pulse. These pulses are rapidly released in a train, following exactly the frequency determined by *V*(*t*). In our simulation experiments we took the following function used in neuroscience for an action potential: either the exponentially decreasing function $F_v(V)={\text {e}}^{-k_s V}$ (see^10,47^), or the alpha function $F_v(V)=V {\text {e}}^{-k_s V}$ (see^48^), where $k_s$ is a positive parameter determining the decay rate.

The parameter $k_a$ is a degradation rate of ACTH and can be obtained from experimental data. The rest of parameters $k_{ah}$, $k_{vh}$, $A_0$, $V_0$ are all positive, they are adjusted empirically to fit the known HPA dynamics.

The structure of Eqs. (1)–(5) seems to be new and develops the mathematical scheme put forward in^5^. Unlike the mathematical formalism of^16^, our Eqs. (1)–(5) are not just differential, but functional-differential and contain impulses (cf.^49^).

(3) The function *H*(*t*) is a synergetic input from the hypothalamic nuclei that combines the CRH drive $H_r(t)$ (from PVN) and the exogenous circadian signal $H_c(t)$ (from SCN).

Following^16^, we neglect a feedback from CORT to the hypothalamus. Thus $H_r(t)$ is defined periodic with a given period $T_r$ and a given degradation rate $k_r$:

7 $$\begin{aligned} H_r(t)={\text {e}}^{-k_r(t-n T_r)},\; nT_r\le t<(n+1)T_r,\quad n\ge 0, \end{aligned}$$

(In a more general model parameters of $H_r(t)$ may be modulated by *C*(*t*) which acts as an inhibitor.)

The signal $H_c(t)$ from the external circadian clock is assumed harmonic and non-negative with the period 24 h (1440 min). For our simulations it is taken

8 $$\begin{aligned} H_c(t)= 0.55 - 0.45 \cos (\omega (t-t_0)). \end{aligned}$$

Here $\omega =2\pi /1440$ is the circadian frequency and $t_0$ shifts the peak of $H_c(t)$ to the time of the expected maximum of circadian activity (cf.^19^).

The period $T_r$ of $H_r(t)$ is usually about an hour, so $H_r(t)$ can be considered high-frequency when compared with the slow circadian drive $H_c(t)$. At a short interval of simulation (of several hours) the circadian input can be considered constant, and (if *H*(*t*) is properly normalized) we can take $H(t)=H_r(t)$ neglecting circadian variations. In a more general situation we consider *H*(*t*) as a product

9 $$\begin{aligned} H(t)= H_r(t)H_c(t), \end{aligned}$$

By this multiplication the two signals are superimposed and the high-frequency signal $H_r(t)$ modulates the low-frequency circadian drive to obtain a cumulative drive *H*(*t*) of a more complex spectrum.

In system (1)–(9) the coordinates *A*(*t*), *C*(*t*) are continuous in time, and *V*(*t*) has jumps at the points $t_n$ defined from functional relationships (4), (5). Some mathematical properties of the system are described in the Supplementary Material.

### Parameters used for simulation

For definitness, assume that *A*(*t*) and *C*(*t*) are measured in pg/ml and ng/ml, respectively, and time is measured in minutes. The parameter values used for computer simulations are presented in Table 1.

**Table 1:** Parameter values used in simulation. The five parameters in the upper part of the table were taken from the experimental data. The rest of the parameters were adjusted to fit the known HPA dynamics.

Parameter	Value	Description
$k_r$	0.028	Degradation rate of CRH
$k_a$	0.7	Degradation rate of ACTH
$k_c$	0.07	Degradation rate of CORT
$T_r$	20	Pulsation period of CRH
$\tau$	15	Delay time in ACTH-induced CORT release
$k_v$	0.005	Leakage rate of *V*
$k_{ah}$	60	Relates to drive from hypothalamus to pituitary
$k_{ca}$	0.05	Relates to drive from pituitary to adrenal gland
$k_{vh}$	0.055	Relates to drive from hypothalamus to *V*
$\Delta$	3.5	Firing threshold for *V*
$A_0$	0.6	Relates to the permanent component of the ACTH pulsation rate
$V_0$	0.05	Minimal action potential
$h_c$	40	Half-range point of repressor function $F_a(C)$
$F_0$	0.1	Minimal value of $F_a(C)$

The first five parameters in Table 1 are taken from^36,50^. Namely, from the supplementary material to^36^, the ranges for the half-life times ($T_{1/2}$) of ACTH and CORT can be taken 0.5–1 min and 7.2–10 min, respectively. (The authors refer to experiments in^23,51^.) The decay rate of an exponentially decreasing signal can be calculated as $\ln (2)/T_{1/2}$. This gives the ranges 0.693–1.386 ${\text {min}}^{-1}$ for $k_a$ and 0.069–0.96 ${\text {min}}^{-1}$ for $k_c$. Following the main text of^36^, we take the delay of $\tau =15$ min. In^50^ the half-life time of CRH is defined as $25.3\pm 1$ min, that gives the decay rate in the range 0.0264–0.0285 ${\text {min}}^{-1}$. (Notice that experimentally found values of hormonal half-lives vary significantly from one publication to another, so this is one of the possible choices.) The rest of the parameters (such as coupling coefficients and gains) were adjusted to achieve the best dynamic fit to the known activity of the HPA axis (cf.^19^).

Notice that the parameters that fit a real date may significantly vary from one human individual to another. This is even more true for various animal species (e.g., rodents), which are usually used in biological experiments. Thus a parametric identification is a challenging problem for this class of models.

## Results

In this section we present some time series plots obtained by computer simulation.

For simulations the MATLAB program for modeling delay differential equations DDE23 was applied^52^. Its event location tool was used to model episodical reset of *V*(*t*). DDE23 uses the the methods of steps. Identical results were obtained when the integration was performed using a 4th-order Runge Kutta algorithm (XPPAUT^53^).

### Simulations for an exponential function $F_v(V)$

Consider the hypothalamic input defined by (9). Let the shape function $F_v(V)= {\text {e}}^{-k_s V}$ with $k_s=0.5$. The circadian input is defined by (8) with $t_0=30$ min. The interval between firings varies from 51 min (at peak) to 74 min (at nadir). For similar experimentally obtained figures see^16^.

**Figure 2:**
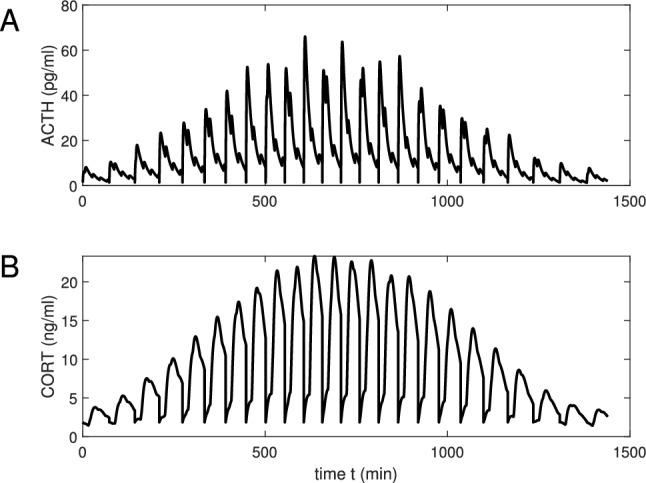
Hormonal profiles for the hypothalamic drive $H(t)=H_r(t)H_c(t)$. The function $F_v(V)$ is taken exponentially decreasing with the decay rate $k_s=0,5$, (**A**,**B**) profile ACTH and CORT plasma concentrations, respectively.

Notice that the hypothalamic oscillator and the ultradian pituitary-adrenal oscillator are coupled in both amplitude and frequency (however the maximum in CORT is phase shifted from that in ACTH). The coupling strength depends on the coefficients $k_{ah}$, $k_{vh}$. The pattern of oscillations for nominal parameters is shown in Fig. 2. It is seen that ACTH and CORT profiles combine at least three rhythms with the periods 20 min, $\sim 60$ min and 1440 min (cf.^35^).

The parameters were chosen to obtain the inter-burst interval close to the value of a physiological ultradian rhythm (that ranges from one hour to two). Note that the fluctuations in CORT are more regular than those observed experimentally (see e.g.^58^). This is a consequence of the simplicity of the integrate-and-fire model we use for pituitary pulsativity. The basic ultradian and the CRH rhythms are superimposed. This may result in an additional complexity of the oscillations spectrum, since ACTH can be secreted not in spikes, but in bursts. Simulations show that this complexity reduces when the ultradian period is a multiple of the CRH period $T_r$. (In this case the ACTH oscillation will be close to periodic with one impulse in the least period.) In other cases ACTH will have multiple impulses in the least period, i.e. bursting is observed. This is consistent with what is known about hormonal secretion in the pituitary^13,43,44,54^. With the increase of the threshold $\Delta$, frequency of the ultradian oscillation decreases. If the threshold is large enough, it is never reached and the system comes to an equilibrium. Note that the maximum in CORT is shifted by 15 min from the maximum in ACTH.

### Simulations for an alpha function $F_v(V)$

Now assume that $F_v(V)=V {\text {e}}^{-k_s V}$ with the decay rate $k_s=0.1$. This case will be used to compare our simulations with some experimental profiles obtained in^45^ for adult male Sprague–Dawley rats. Simulated plots are shown with thick black lines, while experimental results are demonstrated with thin red lines.

**Figure 3:**
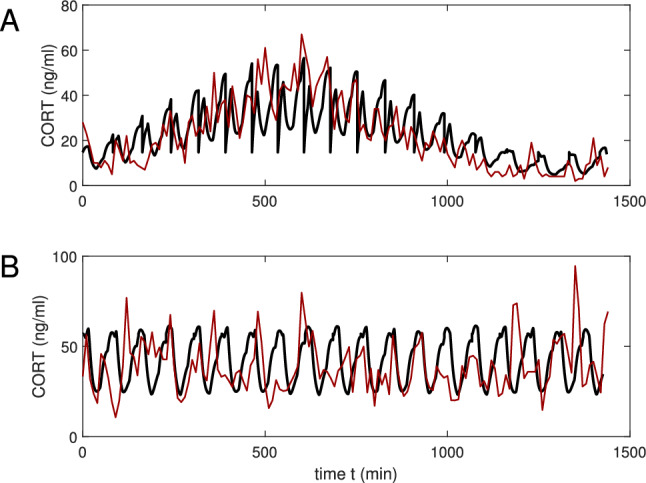
Comparison of model (black lines) with measured corticosterone (CORT) profiles (red lines) in (**A**) healthy rats and (**B**) rats with an electrolytic lesion in the suprachiasmatic nucleus^45^. (**A**) Shows the mean value for 7 healthy rats and (**B**) shows the mean value for 5 lesioned rats (for standard errors of the mean, see^45^). In (**A**) the predicted values of CORT when *H*(*t*) represents circadian periodic drive and in (**B**) $H_r(t)$ for no periodic drive. In both cases $F_v(V)$ is given by an alpha function with $k_s=0.1$.

In Fig. 3A we plot a CORT profile for the circadian hypothalamic drive $H(t)=H_r(t)H_c(t)$ with the circadian peak shifted to $t_0=-80$ min. The simulated interval between firings varies from 71 min (at peak) to 87 min (at nadir). The comparison is made with the mean CORT concentration for the cohort of control (intact) rats (in read).

For Fig. 3B we simplify the dynamics and assume that we can neglect the modulation from the circadian SCN clock. Then the hypothalamic input can be defined not by (9), but as $H(t)=H_r(t)$, where $H_r(t)$ is given by (7). Our modeling result is compared with Figure 3C^45^ experimentally obtained for rats with SCN lesions. Our plot shows more regularity, but generally follows the experimental pattern. The basic ultradian signal has the interpulse period of approximately 76 min.

### Ultradian oscillations in an isolated pituitary

Consider the case when the pituitary gland is isolated from the hypothalamus and the adrenal gland. Here we will model the shape in vitro experiment described in^27^, where seven fetal and two adult human pituitaries were placed in a flow-through perfusion chamber and oxygenated. It was found in^27^ that ACTH still exhibits a pulsatile ACTH release with low amplitudes and high frequencies. Assume that the inequality

10 $$\begin{aligned} \Delta k_v < V_0 \end{aligned}$$

is satisfied, i.e. the firing threshold is sufficiently small, and $H\equiv 0$, $C\equiv 0$. Here it will shown analytically that system (1)–(5) has a globally stable periodic solution. If (10) does not hold, the system solutions approach an equilibrium with the increase of time. Notice that previous mathematical models have not been able to explain this oscillatory phenomenon.

When $H\equiv 0$, $C\equiv 0$, system (1)–(3) is reduced to

11 $$\begin{aligned} {\dot{A}}= -k_a A + A_0\, F_v(V), \end{aligned}$$

12 $$\begin{aligned} {\dot{V}}= -k_v V + V_0, \end{aligned}$$

and Eqs. (4), (5) are the same as previously. It is seen that (12) can be integrated independently of (11). Hence

13 $$\begin{aligned} V(t) = \frac{V_0}{k_v}\, \left( 1 - {\text {e}}^{-k_v (t-t_n)} \right) , \quad t_n<t<t_{n+1}, \end{aligned}$$

and $V(t_{n+1})=\Delta$. Assume that inequality (10) is valid. Then $t_{n+1} - t_n\equiv T$, where

14 $$\begin{aligned} T = - \frac{1}{k_v}\, \ln \left( 1 -\frac{\Delta k_v}{V_0} \right) . \end{aligned}$$

Thus $t_n=t_0+ nT$, $n\ge 0$, and the function *V*(*t*) is *T*-periodic for $t\ge t_0$.

In the case $k_v\rightarrow +0$ inequality (10) is satisfied, so *V*(*t*) is periodic. It follows a saw-tooth pattern

$$\begin{aligned} V(t) = V_0 (t-t_n), \quad t_n<t<t_{n+1}, \quad t_n=t_0+nT, \end{aligned}$$

where $$T=\Delta /V_0$$.

Assume that (10) is valid and consider Eq. (11). Then

15 $$\begin{aligned} A(t) = {\text {e}}^{-k_a(t-t_n)} A(t_n) + A_0 \int _{t_n}^t {\text {e}}^{-k_a(t-s)} F_v(V(s))\,ds, \quad t_n\le t \le t_{n+1}. \end{aligned}$$

Since $V(t+t_n) = V(t+t_0)$ for all $t\ge 0$, from (15) we get a discrete-time relationship

16 $$\begin{aligned} A(t_{n+1}) = {\text {e}}^{-k_a T} A(t_n) + A_0 J_0, \quad n\ge 0, \end{aligned}$$

where

$$\begin{aligned} J_0 = \int _{0}^T {\text {e}}^{-k_a(T-s)} F_v(V(s+t_0))\,ds. \end{aligned}$$

The discrete-time mapping $A(t_n) \mapsto A(t_{n+1})$ defined by (16) has a fixed point

17 $$\begin{aligned} A_* = \left( 1- {\text {e}}^{-k_a T}\right) ^{-1} A_0 J_0, \end{aligned}$$

and for any initial value $A(t_0)$ we have $A(t_n) \rightarrow A_*$ as $n\rightarrow +\infty$. It is easily seen that if we take the initial value $A(t_0)=A_*$, then *A*(*t*) defined by (15) is *T*-periodic.

If (10) does not hold, i.e. $\Delta k_v\ge V_0$, then $V(t)<\Delta$ for all $t\ge t_0$ and the threshold $\Delta$ is never reached. Hence *V*(*t*) has no jumps, it is monotonously increasing for $t\ge t_0$ and

$$\begin{aligned} V(t) \rightarrow V_0/k_v, \quad A(t)\rightarrow A_0F_v(V_0/k_v)/k_a \quad \text{ as }\quad t\rightarrow +\infty , \end{aligned}$$

i.e. the solution tends to an equilibrium.

## Discussion

Our mathematical model captures the main features of the HPA hormonal system, including ultradian and circadian rhythms. Our investigations were mainly inspired by pioneering works^16,42^ and subsequent publications developing this approach. However, unlike these studies, we assume that the CRH drive is not constant, but periodic, with the period less than the ultradian one. Notice that unlike^16^, in our model the time delay is not critical for oscillatory behavior but only introduces a small phase shift in the secretory activity of the corticotrophs. In particular, the delay-free system is also oscillatory. Oscillatory behavior can be lost only in the case of too large values of the threshold $\Delta$ (see Supplementary Material for exact estimates).

In our model the pulsatile exocytosis of ACTH is related to the intrinsic excitability of corticotrophs. Although corticotrophs can generate isolated spikes and bursts, it is the prolonged depolarizations associated with pseudo-plateau bursting that is associated with ACTH exocytosis^13,55^. Large conductance calcium- and voltage-activated potassium (BK) channels are important for this type of bursting^13,56^. An important observation is that, at least in vitro, $>90$% of corticotrophs exhibit only spontaneous spiking activity^55^. Thus it is possible that exocytosis in vivo is related to coordinated dynamic activity in functional networks of corticotrophs^57^. Here we have assumed that bursting in the ACTH release can be explained by imposing a high-frequency CRH rhythm on the intrinsic ultradian rhythm of the ACTH. In this way our model realistically simulates the interplay between the rhythms of the PVN and the anterior pituitary.

In the model we hypothesized the existence of the three oscillation clues related to the hypothalamus and the anterior pituitary. It seems that the dynamics of a hormonal systems depends on the strength of coupling between them. It is assumed that for the HPA axis the coupling between PVN and pituitary (determined by the parameters $k_{ah}$, $k_{vh}$) is rather weak. Thus the intrinsic pulse generator located in the anterior pituitary plays the leading role and determines the main hormonal rhythm. The system’s dynamics is also significantly affected by the drive from the pituitary pulse generator to the adrenal gland (described with the coefficient $k_{ca}$).

Here we combine mathematical tools that were traditionally used in the modeling of endocrine systems, such as ordinary differential equations and delay differential equations, with the mathematical technique of impulsive differential equations used in describing neural activity. Such a synthesis enables explaining impulsive signalling from the brain to endocrine glands in an understandable and convenient way.

The pulses generated by the HPA axis are more irregular than those generated by our simple model (compare, for example,^12,45,58^). Thus, the mechanism for pulse generation must be more complex than that of a simple integrate-and-fire neuron. Indeed, in vivo imaging studies suggest that hormone release by the pituitary gland is coordinated with the microcirculation^59^. In any case, it is clear that the interesting mathematics in the HPA axis occur at the level of the pituitary gland.

Our model describes the function of the HPA axis on a time scale of 24 hours. However, it is known that in conditions of chronic stress and certain psychiatric disorders the HPA axis can become dysregulated over times scales of weeks to months. Recently it has been suggested that this dysregulation arises because of slow changes in the functional masses of the corticotrophs and the adrenal cells, and a relevant mathematical model has been proposed^60^. Applying this effect to our model implies that the parameters $k_{ah}$, $k_{vh}$, $k_{ca}$ can vary in time and follow their own dynamics. Our future research will be directed towards exploring the effects of chronic and acute stress in the context of the simple model we have developed in this study.

## Supplementary Information

Supplementary Information.

## Data Availability

The code of our MATLAB program is available on request.
