# Supplementary material for: Modeling pulsativity in the hypothalamic–pituitary–adrenal hormonal axis
Source: Sci Rep. 2022 May 19;12:8480. doi: 10.1038/s41598-022-12513-w (PMC9120490; doi:10.1038/s41598-022-12513-w)
Supplement: Supplementary file 1 — Supplementary Information. [file 41598_2022_12513_MOESM1_ESM.pdf]

Supplementary material for the manuscript: Modeling pulsativity in the hypothalamic-pituitary-adrenal hormonal axis

Alexander N. Churilov and John G. Milton

Mathematical model

Consider a system of differential equations with delay and impulses:

$$\dot{C} = -k_c C + k_{ca} A(t-\tau), \quad (1)$$
$$\dot{A} = -k_a A + (A_0 + k_{ah} H)\, F_a(C) F_v(V), \quad (2)$$
$$\dot{V} = -k_v V + (V_0 + k_{vh} H)\, F_a(C). \quad (3)$$

The coordinates $C(t)$, $A(t)$ are continuous in time, and the potential function $V(t)$ is left-continuous and may have impulses (jumps). The pulsation times t_n are defined from the recursion

$$t_0 = 0, \quad t_{n+1} = \min\{t \,:\, t > t_n, \quad V(t) = \Delta\}, \quad (4)$$

where Δ is a given firing threshold. After the firing the function $V(t)$ resets to zero:

$$V(t_n^+) = 0. \quad (5)$$

Here $V(t_n^+)$ is the right-sided limit of $V(t)$ at the point t_n.

Assume that $F_a(\cdot)$ and $F_v(\cdot)$ are nonlinear functions,

$$F_a(C) = F_0 + \frac{1}{1 + C/h_c}, \quad F_v(V) = \mathrm{e}^{-k_s V} \quad \text{or} \quad F_v(V) = V\mathrm{e}^{-k_s V}. \quad (6)$$

The parameters k_a, k_c, k_v, k_{ah}, k_{ca}, k_{vh}, A_0, V_0, τ, k_s, h_c, F_0 are all positive.

The function $H(t)$ decribes a synergetic input from the hypothalamic nuclei. In our simulations it is taken periodic or quasiperiodic.

System (1)–(5) is functional-differential with a discrete delay and jumps. Let $t_0 = 0$ be the initial time and $V(t_0^+) = 0$. For any initial data $C(t_0)$, $A(t) = \varphi(t)$, $t \in [t_0 - \tau, t_0]$, where $\varphi(t)$ is a continuous initial function, there exists a unique solution of system (1)–(5).

Simple properties of the model

Equations (1)–(3) can be rewritten as

$$\dot{C}(t) = -k_c C(t) + f_c(t), \quad (7)$$
$$\dot{A}(t) = -k_a A(t) + f_a(t), \quad (8)$$
$$\dot{V}(t) = -k_v V(t) + I(t), \quad (9)$$

where

$$f_c(t) = k_{ca} A(t-\tau), \quad (10)$$
$$f_a(t) = (A_0 + k_{ah} H(t))\, F_a(C(t)) F_v(V(t)), \quad (11)$$
$$I(t) = (V_0 + k_{vh} H(t))\, F_a(C(t)). \quad (12)$$

Proposition 1 *System (1)–(3) is positive, i.e. its solutions have non-negative components for any non-negative initial data. Namely, let the input signal $H(t) \geq 0$ for $t \geq t_0$, $A(t) \geq 0$ for $t_0 - \tau \leq t \leq t_0$ and $C(t_0) \geq 0$. Then $A(t) \geq 0$, $C(t) \geq 0$, $V(t) \geq 0$ for all $t \geq t_0$.*

Proof Obviously $I(t) \geq 0$ and hence $0 \leq V(t) \leq \Delta$ for all $t \geq t_0$. From (7), (8) we obtain

$$C(t) = \mathrm{e}^{-k_c(t-t_0)}C(t_0) + \int_{t_0}^{t} \mathrm{e}^{-k_c(t-s)} f_c(s)\,ds, \tag{13}$$

$$A(t) = \mathrm{e}^{-k_a(t-t_0)}A(t_0) + \int_{t_0}^{t} \mathrm{e}^{-k_a(t-s)} f_a(s)\,ds, \quad t \geq t_0. \tag{14}$$

Then the statement of the proposition evidently follows. □

Further we suppose that the conditions of Proposition 1 are fulfilled, because only positive decisions have biological meaning. Let $0 \leq H^- \leq H(t) \leq H^+$ for $t \geq t_0$, where H^-, H^+ are some numbers. Introduce a number

$$F_v^+ = \begin{cases} 1, & F_v(V) = \mathrm{e}^{-k_s V}, \\ (k_s \mathrm{e})^{-1}, & F_v(V) = V\mathrm{e}^{-k_s V}. \end{cases}$$

Evidently $F_v(V) \leq F_v^+$ for all V.

Proposition 2 *Define*

$$A^+ = (A_0 + k_{ah}H^+)(F_0+1)F_v^+/k_a, \quad C^+ = A^+ k_{ca}/k_c.$$

Then $A(t)$ and $C(t)$ are bounded for $t \geq t_0$, and

$$\limsup_{t\to+\infty} A(t) \leq A^+, \quad \limsup_{t\to+\infty} C(t) \leq C^+.$$

Proof The proof follows from (13), (14). □

Obviously $I^- \leq I(t) \leq I^+$ for all $t \geq t_0$, where

$$I^- = (V_0 + k_{vh}H^-)F_0, \quad I^+ = (V_0 + k_{vh}H^+)(F_0+1).$$

Proposition 3 *Let*

$$I^- > \Delta k_v. \tag{15}$$

Define $T_n = t_{n+1} - t_n$, $n \geq 0$. Then for any solution of (9), (4), (5) *and all $n \geq 0$ we have*

$$-\frac{1}{k_v}\ln\left(1 - \frac{\Delta k_v}{I^+}\right) \leq T_n \leq -\frac{1}{k_v}\ln\left(1 - \frac{\Delta k_v}{I^-}\right). \tag{16}$$

Proof The proof follows immediately from the integral representation

$$V(t) = \int_0^{t-t_n} \mathrm{e}^{-k_v(t-t_n-s)} I(s+t_n)\,ds, \quad t_n \leq t \leq t_{n+1} \tag{17}$$

and from the equality $V(t_{n+1}) = \Delta$. □

In the special case $k_v \to +0$ (a perfect integrator) (15) is satisfied and (16) takes the form

$$\Delta/I^+ \leq T_n \leq \Delta/I^-. \tag{18}$$

If $I^- < \Delta k_v$, then the threshold Δ is never reached and the function $V(t)$ has no jumps.
